# Multiplex Lateral Flow Immunoassay for the Detection of Expanded-Spectrum Hydrolysis and CTX-M Enzymes

**DOI:** 10.3390/diagnostics12010190

**Published:** 2022-01-13

**Authors:** Christian Moguet, Camille Gonzalez, Thierry Naas, Stéphanie Simon, Hervé Volland

**Affiliations:** 1 Université Paris Saclay, CEA, INRAE, Département Médicaments et Technologies pour la Santé (DMTS), SPI, 91191 Gif-sur-Yvette, France; christian.moguet@cea.fr (C.M.); stephanie.simon@cea.fr (S.S.); 2 Bacteriology-Hygiene Unit, APHP, Hôpital Bicêtre, 94270 Le Kremlin-Bicêtre, France; gonzalezcamille0405@gmail.com (C.G.); thierry.naas@aphp.fr (T.N.); 3 Team Resist, UMR1184, Université Paris-Saclay—INSERM—CEA, LabEx Lermit, 92296 Châtenay-Malabry CEDEX, France; 4 Associated French National Reference Center for Antibiotic Resistance: Carbapenemase-Producing Enterobacteriaceae, 94270 Le Kremlin-Bicêtre, France

**Keywords:** lateral flow immunoassay, β-lactamase detection, CTX-M enzymes, cephalosporin hydrolysis

## Abstract

Background: Early detection of expanded-spectrum cephalosporinase (ESC) hydrolyzing ß-lactamases is essential for antibiotic stewardship. Here we have developed a multiplex lateral flow immunoassay (LFIA) that detects cefotaxime-hydrolyzing activity as well as the most prevalent ESC-hydrolyzing ß-lactamases: the CTX-M-like. Methods: The Rapid LFIA ESC test was evaluated retrospectively on 188 (139 Enterobacterales, 30 *Pseudomonas* spp. and 14 *Acinetobacter* spp.) agar-grown bacterial isolates with well-characterized ß-lactamase content. One single colony was resuspended in 150 µL extraction buffer containing cefotaxime, incubated at room temperature for 30 min prior to loading on the LFIA for reading within 10 min. Results: Out of the 188 isolates, all 17 that did not express a β-lactamase hydrolyzing cefotaxime gave negative results, and all 171 isolates expressing a β-lactamase known to hydrolyze cefotaxime, gave a positive test result. In addition, all 86 isolates expressing a CTX-M-variant belonging to one of the five CTX-M-subgroups were correctly identified. The sensitivity and specificity was 100% for both tests. Conclusions: The results showed that the multiplex LFIA was efficient, fast, low cost and easy to implement in routine laboratory work for the confirmation of ESC hydrolyzing activity and the presence of CTX-M enzymes.

## 1. Introduction

The fight against bacterial resistance to antibiotics is becoming one of the greatest challenges facing our society, especially with the spread of multidrug-resistant strains [1]. β-Lactams are among the most frequently prescribed antibiotics to treat bacterial infections due to their safety and clinical efficacy. However, with the global proliferation of β-lactamases (BL), a resistance mechanism commonly found in Gram-negative bacteria (GNB), particularly Enterobacterales, their usefulness is under threat [2,3]. In healthcare settings, Enterobacterales producing extended-spectrum β-lactamases (ESBL) are a particular concern. The CDC has estimated that these Enterobacterales account for 19% of healthcare-associated infections each year and that infections caused by these bacteria are also associated with increased mortality and cost of care [4]. ESBLs have limited treatment options because they hydrolyse most β-lactams, including penicillin and extended-spectrum cephalosporins (ESCs) (e.g., cefotaxime [CTX] and ceftazidime [CAZ]), and carry many other resistance determinants leading to a multidrug-resistant phenotype [2]. Their spread is of great clinical concern given the importance of these pathogens as causes of hospital-acquired and community-acquired infections, resulting in difficult-to-treat infections associated with high mortality rates and increased costs [5].

Within the ESBLs, the CTX-M family emerged in the early 1980s and now represents the most common ESBLs in the world. It is divided into five groups based on amino acid sequence identity: the CTX-M groups 1; 2; 8; 9 and 25 [6,7]. Each group comprises allelic variants that differ from each other by one or more amino acid substitutions [5,8]. The CTX-M-15 variant of CTX-M Group 1 is considered the most common in many parts of the world. However, it is supplanted by CTX-M Group 9 variants in China, Japan, South-East Asia and Spain, and in particular the CTX-M-14 variant [7]. Infections caused by CTX-M-producing bacteria must be treated by carbapenems. This has led to the increasing use of carbapenems, followed by the emergence and spread of carbapenemase-producing strains [7,9]. CTX-M expressing Enterobacteriaceae are associated with higher rates of healthcare costs, morbidity and mortality [10,11].

Rapid and efficient detection of antibiotic-resistant bacteria is an essential step for responsible antibiotic stewardship and infection control, and there are currently several diagnostic tests available for the identification of CTX-M carriers in the clinical setting.

They are either based on genotypic or phenotypic approaches [12,13]. Genotypic methods mainly use nucleic acid amplification, which remains the reference method for the detection of ESBLs including CTX-Ms [14,15,16,17,18]. Many of these tests are home-made PCR tests [14,16], but several tests are commercially available for the detection of CTX-M-like [15]. However, these methods require specific equipment, trained personnel and are usually expensive. In addition, the appearance of new variants may not be detected by these techniques due to changes in nucleotide sequences [12].

In contrast to genotypic methods, which are based on the identification of the resistance gene, phenotypic methods reveal the presence of enzymatic activity and in some cases its identification for immunochromatographic lateral flow immunoassay (LFIA) testing. There are many phenotypic methods, each with its own drawbacks: disc diffusion tests which require 24 h of incubation [13]; colorimetric tests (β-Lacta [19]; ESBL NDP [20]) where the interpretation of the colour change can make it difficult to read the results; matrix-assisted laser desorption time-of-flight mass spectrometry (MALDI-TOF MS) requiring expensive equipment and significant expertise [21,22]; UV spectrophotometry, reserved for reference laboratories due to its complexity [23]; and direct β-lactam inactivation method (dBLIM), which allows the detection of extended-spectrum cephalosporinase activity directly from Enterobacterales under inactivation conditions, but is often limited by the time required to obtain results and undetermined complementary mechanisms [24]. Indeed, despite technological improvements, the identification of pathogenic bacteria and the detection of antibiotic resistance remains complex and time consuming [25]. However, in the past decade, the development of LFIA for antibiotic resistance has improved diagnosis in the identification of BLs.

LFIAs have proven useful as easy, rapid and reliable confirmatory tests for the detection of antibiotic resistance mechanisms, particularly for BLs in Gram-negative bacteria [26,27,28]. The NG-Test CTX-M MULTI showed 100% sensitivity and specificity with isolates grown on agar plates and was able to detect 98% of ESBL producers identified in a clinical setting in France, either from colonies or from positive blood cultures [27]. These tests detect the presence of enzymes that confer resistance to ESBLs, and although CTX-Ms represent most of the resistance mechanisms leading to ESC resistance, some rare enzymes may be missed (plasmid-encoded AmpC, hyper AmpCs and some carbapenemases). In parallel, the LFIA-CTX test showed 99.1% sensitivity and 100% specificity for the detection of ESC hydrolytic activity from colonies [29]. Recently, an evaluation was carried out on three biochemical tests for ESC hydrolysis: the ESBL NDP test, the β-Lacta^TM^ test and the LFIA-CTX test, combined with the NG-test CTX-M-Multi. The LFIA-CTX test showed better sensitivity and specificity than the other tests, particularly for Enterobacterales. Furthermore, unlike the ESBL NDP test, the LFIA-CTX is not limited to detection of ESBL. Thus, it was concluded that the combination of LFIA-CTX with NG-CTX-M-Multi would detect hydrolysis of ECS as well as the most frequently encountered ESBLs.

In this study, we have developed a single LFIA strip, named LFIA Rapid ESC, which combines the LFIA-CTX and NG-CTX-M-Multi tests, and allows the identification of CTX-M-like (98% of ESBLs) and the detection of ESC hydrolysis directly from colonies grown on different culture media widely used in clinical microbiology laboratories.

## 2. Experimental Section

### 2.1. Monoclonal Antibodies

Monoclonal antibodies (mAbs) were obtained as described in two previous articles by this group [27,29] and were adapted into a single LFIA strip.

### 2.2. Manufactured Test

The required mAbs were produced on a large scale for the development of the LFIA Rapid ESC test. The manufacture of the strips has been described previously [29].

The LFIA Rapid ESC test has three capture lines present on the nitrocellulose membrane. The first line (LT1) was prepared by immobilizing cefotaxime-BSA (0.1 mg/mL). For the second line (LT2), the three capture mAbs for CTX-M were pooled and immobilized (0.5 mg/mL for each mAb). These mAbs allow the detection of all 5 CTX-M groups due to the cross-reactivity of some mAbs with the other two groups (Groups 8 and 25). The third line, the control (LC), was prepared with goat anti-mouse mAbs (1 mg/mL).

The detection mAbs were coupled to colloidal gold. The amounts of tracer before deposition on the conjugate paper for the anti-cefotaxime mAb was OD_600nm_ 0.9 and for the anti-CTX-Ms the OD6_00nm_ was 1.2. The minimum amount of intact cefotaxime used to inhibit any signal on LT1 was 15 ng/mL.

**Figure 1:**
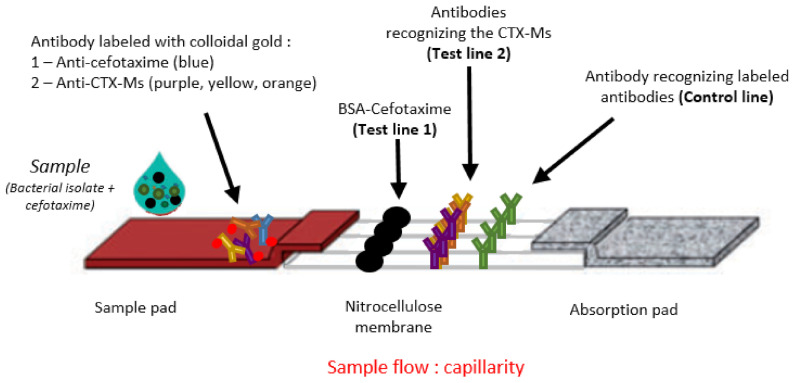
Structure of the strips. The strip consists of the sample pad with dried gold-labelled mAbs (anti-cefotaxime + anti-CTX-Ms); the nitrocellulose membrane with a Test Line 1 consisting of intact CTX coupled with BSA; a Test Line 2 consisting of mAbs recognizing CTX-Ms; a control line consisting of mAbs recognizing the colloidal gold-labelled mAbs; and the absorption pad which allows the sample to migrate along the strip.

**Figure 2:**
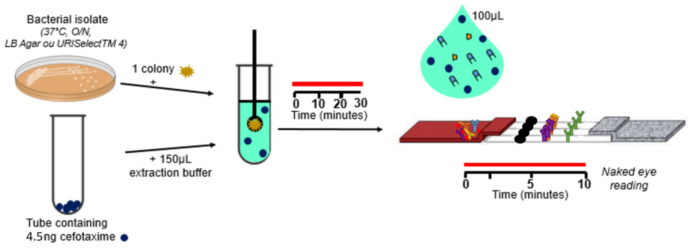
Test procedure. One colony is resuspended in 150 µL of extraction buffer with 15 ng/mL of CTX. After a 30-min incubation at room temperature (RT), 100 µL are loaded on the cassette. The results are obtained after 10 min of migration. Data acquisition is by naked eye readings.

The strips were then enclosed in a plastic cassette to facilitate test execution. The structure of the strip is illustrated in Figure 1, and the test procedure in Figure 2.

### 2.3. Assay Protocol for Test Validation

The strains to be tested were grown overnight at 37 °C on URISelectTM 4 (Bio-Rad, Marnes la Coquette, France). One single isolated colony was collected from the plate with a 1 µL inoculation loop and suspended in 150 µL of extraction buffer (Tris-HCl 0.1 M pH 8.0, NaCl 0.15 M, BSA 0.1%, Tween 20 0.5%, CHAPS 1%, sodium azide 0.01%) containing 15 ng/mL of CTX. The extraction buffer allowed bacterial lysis. After 30 min incubation, 100 µL of this extract was loaded on the strip and allowed to migrate for 10 min.

### 2.4. Data Acquisition

Data were acquired by naked eye readings. The CTX-M BL activities that hydrolyzed CTX were identified when a signal appeared on Test Line 1 and 2. Other BL activities that hydrolyzed CTX were identified with a signal only on Test Line 1 (see Figure 2). The control line had to be present to validate the LFIA test.

### 2.5. Strains Tested

A total of 188 isolates with PCR-characterized BL content were used to validate the LFIA test, which included a variety of bacterial species: Enterobacterales (*E. coli*, *K. pneumoniae*, *E. cloacae*, *E. asburiae*, *C. freundii*, *C. koserii*, *M. morganii*, *Salmonella* spp., *and S. marcescens*) and non-fermenters (*A. baumanii*, *P. aeruginosa*). This collection included 17 isolates unable to hydrolyze cefotaxime including one strain that expressed CTX-M-93 and a CTX-M-variant lacking CTX activity, and 171 isolates known to express CTX hydrolyzing enzymes including 85 CTX-M-producing isolates.

## 3. Results

### 3.1. Test Interpretation

**Figure 3:**
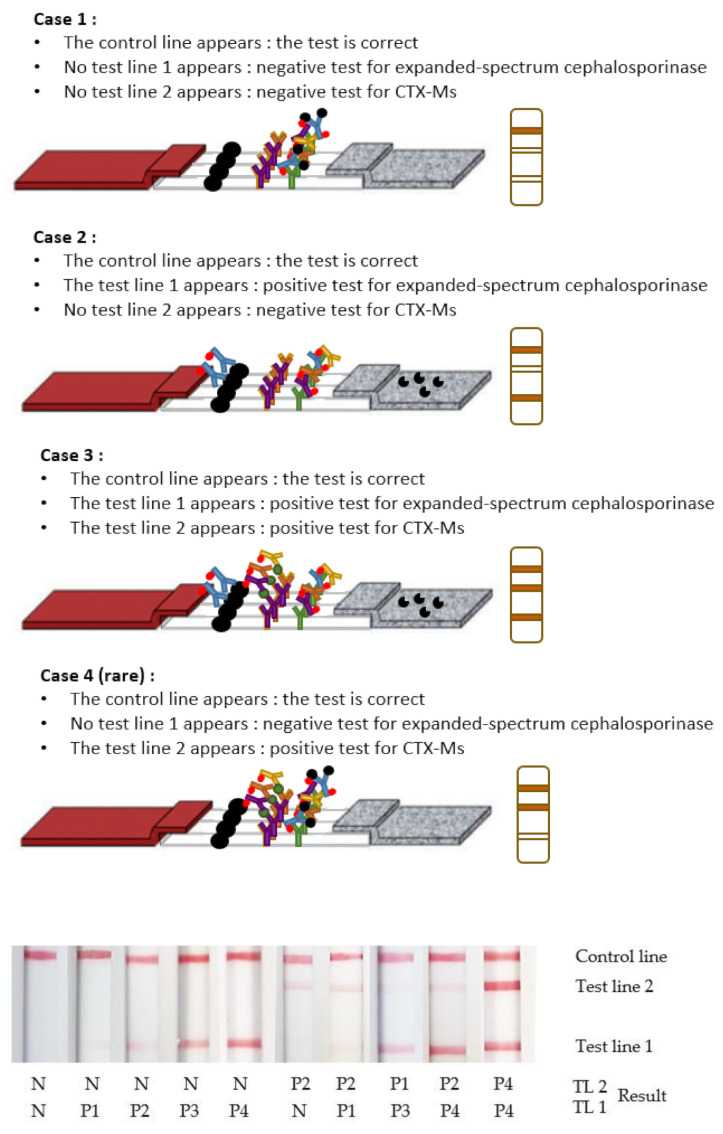
Test interpretation. Case 1: In the absence of enzymatic activity, all the anti-cefotaxime mAb’s paratopes are occupied by the CTX added to the sample before the test. On both tests lines, the results are negative: “N”. The control line is positive: “P”. Case 2: In the presence of enzymatic activity other than CTX-Ms, the hydrolysed CTX is not recognized by the anti-cefotaxime mAbs, which are able to react with the CTX immobilized on the Test Line 1. A signal appears only on the Test Line 1 and the control line “P”, when cephalosporinase expressing strains, other than CTX-Ms, are present. Case 3: In the presence of CTX-Ms, the hydrolysed CTX is not recognized by the anti-cefotaxime mAbs, which are able to react with the CTX immobilized on the Test Line 1. The labelled anti-CTX-Ms mAbs fix CTX-Ms and react with the anti-CTX-Ms mAbs on the Test Line 2. A signal appears on the Test Line 1, the Test Line 2 and the control line “P”. Case 4: In rare cases with some cephalosporinase-expressing strains such as CTX-M-93, the CTX is not hydrolysed. All the paratopes of anti-cefotaxime mAbs are then occupied by the CTX added to the sample before the test. The labelled anti-CTX-Ms mAbs fix CTX-Ms and react with the mAbs on the Test Line 2. A signal appears only on the Test Line 2 and the control line “P”. The associated number of “P” is relative to the intensities observed on the test lines.

Depending on the BL(s) involved in the enzymatic reaction, the test lines and their interpretation differ. These are described in Figure 3, with examples of results obtained from a cassette.

### 3.2. Test Validation

For the LFIA validation, 188 isolates with PCR-characterized BLs content were tested (Table 1 and Table 2). Detailed results are available in Supplementary Table S1.

The 17 isolates unable to hydrolyze CTX gave negative results for CTX hydrolysis activity (see Table 1). The majority of these isolates either did not express any ß-lactamase, expressed a narrow spectrum ß-lactamase or a basal chromosome-encoded AmpC that did not hydrolyze CTX significantly. OXA-21 is a penicillinase with no or very low enzyme activity for ESC including CTX. IMI-1/-2/-7 and OXA-198 are Class A and D carbapenemases, respectively, that do not hydrolyze ESCs. As expected, CTX-M-93, that is unable to hydrolyze CTX, gave a positive signal only on Test Line 2, which reveals the presence of a CTX-M-like ß-lactamase.

All of the 171 strains able to hydrolyze CTX (Table 2) gave a positive signal on Test Line 1 (see Table 2). All overexpressed-AmpCs, ESBLs (SHV, TEM, CTX-Ms, etc.) and the majority of carbapenemases (KPC, NDM, VIM, IMP and OXA) could be detected by the test, as has been described previously [29]. In addition, irrespective of species, the 86 strains expressing CTX-M gave a positive signal on Test Line 2. One *E. coli* isolate expressing CTX-M-93 was unable to hydrolyze CTX, but 85/86 isolates that expressed CTX-M enzymes displayed a CTX-hydrolytic activity.

### 3.3. Overall Performance

The results obtained during this validation showed that our LFIA Rapid ESC has a sensitivity and specificity of 100% for the detection of CTX hydrolytic activity and for the identification of CTX-M-like enzymes. This allows the detection of ESC hydrolytic activity at the same time as the most prevalent resistance mechanism responsible for ESC hydrolysis.

## 4. Discussion

The spread of GNB isolates expressing BLs capable of hydrolysing ESC is a major public health concern [7] leading to increased morbidity and mortality rates in humans and escalating financial costs. Rapid identification of these isolates is essential to adapt treatment in case of infection, but also to prevent their spread in the hospital environment by implementing infection control measures. The earlier the enzyme activity is detected, the more effective the treatment and the lower the probability of nosocomial spread [30]. Among GNB with BL ESC activity, CTX-M enzymes currently represent the most widespread group of ESBLs, with more than 246 CTX-M variants described [7,31]. CTX-Ms displays significant enzymatic activity against ESC that result in therapeutic treatment failure, and are the main source of ESC resistance in Enterobacterales isolated from humans.

The LFIA Rapid ESC multiplex assay was able to identify CTX-M enzymes, which represent a large proportion of ESBLs (e.g., 98% of ESBLs in France [27]), as well as detect most ESC hydrolysing enzymes (other ESBLs, chromosomally and plasmid encoded-AmpCs and most carbapenemases). This wide detection spectrum is due to the use of CTX, which is hydrolyzed by the vast majority of ESBLs, plasmid-encoded or hyper AmpCs, and carbapenemases; ceftazidime, in contrast, is only weakly hydrolysed by these enzymes. Thus, the few enzymes that might be missed by the NG-Test CTX-M-multi will be revealed by the detection of enzymatic activity on CTX.

After a 30-min incubation, positive results were interpretable within 10 min of migration. The LFIA Rapid ESC test was validated on Enterobacterales (*n* = 139), *Pseudomonas* spp. (*n* = 30) and *Acinetobacter* spp. (*n* = 19). All known CTX hydrolyzing BLs in this study as well as those belonging to CTX-M groups were correctly detected and identified within 10 min. In our study, 45.7% of the isolates contained CTX-M enzymes, 6.3% SHV enzymes, 2.12% TEM enzymes, 3.1% GES enzymes, 4.7% chromosomal or plasmid AmpCs, and 19.1% carbapenemases. The remaining isolates tested contained at least two BLs (combination of AmpC, ESBL and/or carbapenemases). No false positive or negative results were obtained. Therefore, the analytical performance of the LFIA Rapid ESC test reached a sensitivity of 100% and a specificity of 100%. These results are consistent with previous studies conducted on both LFIA tests independently, namely the LFIA-CTX and the commercially available NG-Test CTX-M-multi [27,29].

The possibility of enzyme extraction at one of the steps in the test allows their activity to be detected even if, as in some bacteria (e.g., *Pseudomonas* spp., *Acinetobacter* spp.), the presence of complementary mechanisms [32] (e.g., reduced outer membrane permeability, production of efflux systems), may decrease the hydrolysis of the antibiotic and thus its detection under normal conditions.

A further advantage of the test is that it can distinguish between susceptible bacteria that naturally produce AmpC and resistant bacteria that overproduce AmpC [33]. AmpCs are usually expressed at an inducible but basal level, which does not lead to significant CTX hydrolysis, but in case of overexpression, they may lead to ESC such as CTX. AmpCs may also be plasmid-encoded (pAmpCs, e.g., DHA and CMY), which results in increased copy number and may also lead to ESC resistance. The prevalence of pAmpCs is still low in many countries compared to ESBL [33]. The BLs of greatest clinical concern are the ESBLs and carbapenemases. ESBLs can be divided into two groups based on their prevalence: major ESBLs (TEM, SHV, and CTX-M) and minor ESBLs (GES, PER, VEB, etc.) [34]. CTX-M ESBLs are now the most prevalent ESBLs worldwide. More than 246 variants have been described belonging to five distinct subgroups of enzymes, with CTX-M-15 and to a lesser extent CTX-M-14 being the most common [31]. For carbapenemases, all five major enzymes (e.g., NDM; IMP; VIM; KPC and OXA) have been detected, even OXA-48 variants which have very low hydrolytic activity on CTX [22,35,36,37]. In a previous study [29], 14/17 (82.3%) OXA-48 producing strains were detected. It should be noted that in 80% of cases, OXA-48 is associated with ESBLs that efficiently hydrolyse CTX (https://maquette.cnr-resistance-antibiotiques.fr/ressources/pages/Rapport_CNR_2018v1.pdf, accessed on 1 January 2022), T. Naas personal comm).

Thus, the LFIA Rapid ESC test allows the detection of ESC hydrolysis linked to a broad range of BLs (overexpressed AmpCs, plasmid-encoded AmpCs, ESBLs and carbapenemases), expressed in a wide variety of bacteria, as well as the presence of CTX-M enzymes. There are currently several other tests available for the detection of ESBLs at the genetic (PCR, sequencing) and phenotypic level (MALDI-TOF MS, β-Lacta, Rapid ESBL NDP test, dBLIM), as described in the first part of the article. The LFIA Rapid ESC test overcomes some of the drawbacks associated with these tests, allowing reliable and rapid identification of CTX-M enzymes (representing 98% of ESBLs currently circulating in France) and detection of hydrolytic activity on ESCs, with results delivered in 40 min. The test is inexpensive and easy to implement in the routine laboratory setting. In addition, the performance of the test has demonstrated very good sensitivity and specificity, making it a preferred tool for resistance diagnosis in bacterial colonies.

## 5. Conclusions

The LFIA Rapid ESC test is fast and easy to use in a clinical microbiology laboratory setting for the routine detection of BLs hydrolyzing third generation cephalosporins and the detection of CTX-M enzymes. It could replace existing tests in countries with limited resources and/or a high prevalence of BLs. The low cost of the test will also allow it to be used in other industries such as food, animal medicine, and the environment. The next steps will be to use it for the detection of expanded-spectrum hydrolysis and CTX-M enzymes in biological matrices (e.g., urine, feces, and blood culture).

## Figures and Tables

**Table 1:** LFIA Rapid ESC test results for the 17 isolates with no CTX hydrolyzing activity.

	Acquired Enzymes	Positive Results (LT1)	Positive Results (LT2)
Enterobacterales (*n* = 12)		0/12	1/12
*Escherichia coli* (*n* = 3)	WT (2); CTX-M-93	0/3	1/3 *
*Klebsiella pneumonia (n = 1)*	WT	0/1	0/1
*Citrobacter freundii* (*n* = 1)	WT	0/1	0/1
*Enterobacter* spp. (*n* = 3)	IMI-1/2/17 (3)	0/3	0/3
*Salmonella* spp. (*n* = 3)	WT (3)	0/4	0/4
*Morganella morganii* (*n* = 1)	WT	0/2	0/2
***Pseudomonas*** **spp. (*n* = 4)**	WT; Overexpressed efflux pumps (2); OXA-198	**0/4**	**0/4**
***Acinetobacter baumannii*** **(*n* = 1)**	OXA-21	**0/1**	**0/1**

* A bacterial isolate containing CTX-M-93.

**Table 2:** LFIA Rapid ESC test results with 171 isolates with CTX-hydrolyzing activity.

	Acquired Enzymes	Positive Results (LT1)	Positive Results (LT2)
Enterobacterales (*n* = 127)		127/127	83/127
AmpCs (*n* = 8)	Overexpressed AmpC (4); DHA-1/2 (2); ACC-1; CMY-136	8/8	0/8
ESBLs (*n* = 81)	TEM-3/24 (3); GES-6; SHV-2a/11/12 (3); CTX-M-1/2/3/8/10/14/15/17/18/19/24/27/32/37/55/57/65/71/82/100/101/182 (68); TEM + SHV (2); TEM-52 + CTX-M-15; SHV-2a + CTX-M-15; OXA-48-like (2)	81/81	70/81
Carbapenemases (*n* = 16)	KPC-2/3 (5); IMP-1/8/14 (3); VIM-2; OXA-48-like (3); NDM-1; TMB-1; GIM-1; FRI-1	16/16	0/16
AmpCs + ESBLs (*n* = 1)	Overexpressed AmpC + CTX-M-15	1/1	1/1
AmpCs + carbapenemases (*n* = 3)	CMY-13 + VIM-1; CMY-4 + OXA-204; Overexpressed AmpC + SME-1	3/3	0/3
ESBLs + carbapenemases (*n* = 16)	SHV-5/12 + IMP-1/8 (3); SHV-5 + VIM-1; SHV-5/11 + OXA-48-like (2); CTX-M-15 + KPC-3; CTX-M-15 + NDM-7/19 (2); TEM-3 + CTX-M-15 + NDM-19; CTX-M-1/9/15 + OXA-48-like (5); SHV-12 + CTX-M-15 + GES-5	16/16	10/16
AmpCs + ESBLs + carbapenemases (*n* = 2)	CTX-M-15 + CMY-4 + OXA-48-like; CTX-M-15 + CMY-6 + NDM-4	2/2	2/2
***Pseudomonas*** **spp.** **(*n* = 26)**		**26/26**	**1/26**
ESBLs (*n* = 8)	TEM-4; SHV-2a; GES-2/9 (2); PER-1/2 (2); OXA-14; CTX-M-2	8/8	1/8
Carbapenemases (*n* = 16)	GES-5; KPC-2; NDM-1; IMP-1/2/13/15/19/26/31/56/63/71 (10); VIM-1/2/4 (3)	16/16	0/16
Broad spectrum penicillinases + AmpCs (*n* = 2)	Overexpressed AmpC + OXA-13/32 (2)	2/2	0/2
***Acinetobacter*** **spp.** **(*n* = 18)**		**18/18**	**1/18**
AmpCs (*n* = 1)	Overexpressed AmpC	1/1	0/1
ESBLs (*n* = 7)	SHV-5 (2); GES-11/12/14 (3); PER-1; CTX-M-15	7/7	1/7
Carbapenemases (*n* = 4)	NDM-1/2 (2); IMP-1/4 (2)	4/4	0/4
AmpCs + carbapenemases (*n* = 6)	Overexpressed AmpC + OXA-23/51/58/97/143/253 (6)	6/6	0/6

## Data Availability

For details on the data leading to the results, refer to the supplementary data, Table S1: results of 188 isolates for validation of the LFIA Rapid ESC test.

## Supplementary Materials

The following supporting information can be downloaded at: https://www.mdpi.com/article/10.3390/diagnostics12010190/s1, Table S1: Results of 188 isolates for validation of the LFIA Rapid ESC test.
